# Sending signals from shoot to root: OPT3 mediates systemic iron and copper signaling in Arabidopsis

**DOI:** 10.1093/plcell/koad052

**Published:** 2023-02-28

**Authors:** Sophie Hendrix

**Affiliations:** Assistant Features Editor, The Plant Cell, American Society of Plant Biologists, USA; Centre for Environmental Sciences, Hasselt University, Diepenbeek, Belgium

Plants have a love-hate relationship with iron (Fe) and copper (Cu). Due to their redox-active nature, these metals are indispensable as cofactors for a wide range of enzymes and crucial for photosynthesis and respiration, among others. However, Fe and Cu can catalyze the formation of highly reactive hydroxyl radicals, posing a serious threat to plant cells if left unchecked. Hence, plants rely on various mechanisms to control intracellular concentrations of these metals and regulate their uptake to make sure cellular Cu and Fe demands are met while avoiding toxicity (Puig et al. 2007). Furthermore, metal uptake via roots is tightly coordinated with metal concentrations in shoots. In this context, OLIGO PEPTIDE TRANSPORTER 3 (OPT3) was shown to play a key role in systemic signaling of Fe deficiency in Arabidopsis (*Arabidopsis thaliana*) (Mendoza-Cózatl et al. 2014; Zhai et al. 2014).

In this issue of The Plant Cell, **Ju-Chen Chia and colleagues** (Chia et al. 2023) reveal that OPT3 is also involved in the maintenance of Cu homeostasis (see Figure). They showed that mature leaves of an Arabidopsis *opt3* mutant contained significantly higher Fe and Cu levels compared to those of wild-type (WT) plants. Within *opt3* leaves, metal accumulation had shifted from the phloem to the xylem and fascicular cambium, and Cu concentrations were significantly lower in the phloem of the *opt3* mutant compared to the WT, suggesting the involvement of OPT3 in Fe and Cu loading into the phloem. Furthermore, the authors showed that OPT3 mediates Cu uptake in heterologous systems. As Cu concentrations in the roots, young leaves, and seed coats of developing seeds were also lower in the *opt3* mutant, they hypothesized that OPT3 contributed to Cu redistribution from source to sink tissues.

**Figure:**
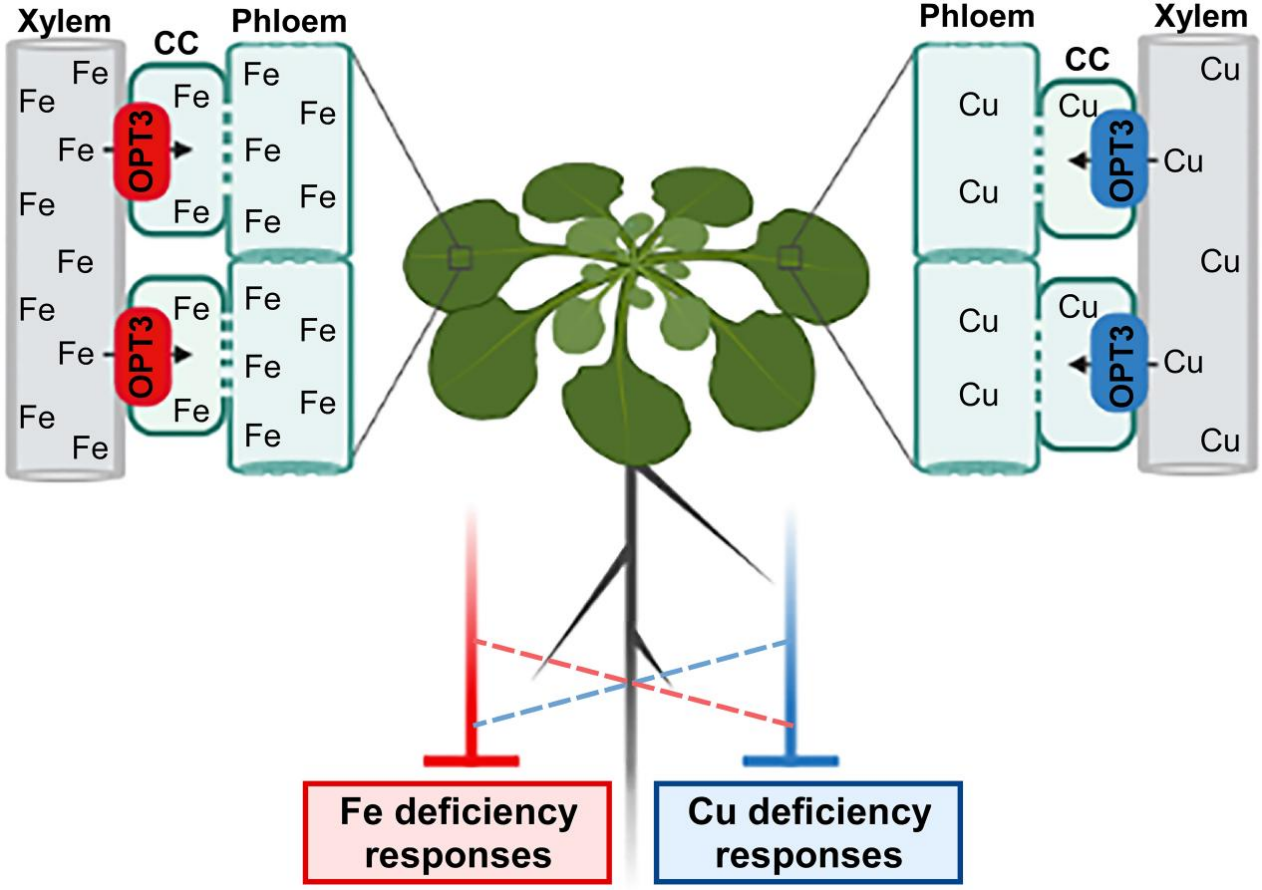
Arabidopsis OPT3 loads copper (Cu) and iron (Fe) into phloem companion cells (CC) for distribution to sink tissues and systemic signaling of Cu and Fe deficiency. Credit: J.-C. Chia and O.K. Vatamaniuk created the figure with BioRender.

Next, they showed that the *opt3* mutant was more sensitive to Cu deficiency and that its roots and young leaves expressed typical Cu deficiency-responsive genes to higher levels compared to those of WT plants, even when grown under Cu-sufficient conditions. These symptoms were rescued by transferring the plants to a nutrient solution with a higher Cu concentration. Although mature leaves of *opt3* contained higher Fe and Cu levels compared to WT plants, *opt3* roots showed a transcriptional Fe and Cu deficiency response, supporting a role for OPT3 in systemic signaling of not only Fe but also Cu. This hypothesis was supported by reciprocal grafting experiments, showing that functional OPT3 in shoots is sufficient to regulate transcriptional Cu and Fe deficiency responses in roots. Interestingly, feeding either Cu or Fe via the shoot phloem partially mitigated both Cu and Fe deficiency responses in *opt3* roots. In addition, feeding Cu- or Fe-deficient WT seedlings with Cu or Fe via the phloem in leaves downregulated the expression of both Cu and Fe deficiency marker genes in roots. These data support the existence of shoot-to-root Cu signaling and suggest that Cu and Fe can partially mimic each other's functions in shoot-to-root signaling via OPT3, adding to the complexity of Cu-Fe crosstalk.

To unravel the interaction between these metals, the authors exposed WT plants to Cu deficiency and Fe deficiency individually or simultaneously. Cu deficiency enhanced Fe accumulation in both roots and shoots. Interestingly, Fe levels were higher in WT plants grown under combined Cu and Fe deficiency than in plants grown under Fe deficiency alone. Furthermore, exposure of WT plants to high Fe concentrations in the growth medium resulted in lower Cu accumulation in roots and shoots. Based on these results, the authors propose that Fe uptake is stimulated in the *opt3* mutant as a consequence of low Cu and Fe concentrations in the shoot phloem and low Cu concentrations in roots. The resulting Fe overaccumulation in roots might in turn negatively affect Cu uptake.

Taken together, this work revealed that besides its role as an Fe transporter, OPT3 contributes to Cu loading into the phloem and mediates systemic signaling of Cu and Fe status from source to sink tissues. How Cu and Fe specifically transduce such signals remains a topic for further study. The knowledge obtained in this study can benefit the development of strategies to boost crop yield on Cu- and Fe-deficient soils and enhance Cu and Fe levels in edible parts of plants to improve their nutritional value.

## References

[koad052-B1] Chia J-C , Yan J, Rahmati Ishka M, Faulkner MM, Simons E, Huang R, Smieska L, Woll A, Tappero R, Kiss A, et al Loss of OPT3 function decreases phloem copper levels and impairs crosstalk between copper and iron homeostasis and shoot-to-root signaling in *Arabidopsis thaliana*. Plant Cell. 2023:35(6):2157–2185. 10.1093/plcell/koad053 PMC10226573 36814393

[koad052-B2] Mendoza-Cózatl DG , Xie Q, Akmakijan GZ, Jobe TO, Patel A, Stacey MG, Song L, Demoin DW, Jurisson SS, Stacey G, et al OPT3 Is a component of the iron-signaling network between leaves and roots and misregulation of OPT3 leads to an overaccumulation of cadmium in seeds. Mol Plant. 2014:7(9): 1455–1469. 10.1093/mp/ssu067 24880337 PMC4153440

[koad052-B3] Puig S , Andrés-Colás N, García-Molina A, Peñarrubia L. Copper and iron homeostasis in *Arabidopsis*: responses to metal deficiencies, interactions and biotechnological applications. Plant Cell Environ. 2007:30(3): 271–290. 10.1111/j.1365-3040.2007.01642.x 17263774

[koad052-B4] Zhai Z , Gayomba SR, Jung H-I, Vimalakumari NK, Piñeros M, Craft E, Rutzke MA, Danku J, Lahner B, Punshon T, et al OPT3 Is a phloem-specific iron transporter that is essential for systemic iron signaling and redistribution of iron and cadmium in Arabidopsis. Plant Cell. 2014:26(5): 2249–2264. 10.1105/tpc.114.123737 24867923 PMC4079381

